# Evolving Inguinal Hernia Repair Practice at the Veterans Health Administration

**DOI:** 10.1001/jamasurg.2026.0307

**Published:** 2026-03-18

**Authors:** Emma M. Bradley, Kathryn A. Schlosser, Michael E. Matheny, Richard A. Pierce

**Affiliations:** 1 Vanderbilt University Medical Center Department of Surgery, Nashville, Tennessee; 2 Tennessee Valley Healthcare System VA Department of Surgery, Nashville; 3 Tennessee Valley Healthcare System VA Geriatric Research Education and Clinical Care Service, Nashville; 4 Beth Israel Deaconess Hospital, Plymouth, Massachusetts; 5 Vanderbilt University Medical Center Department of Biomedical Informatics, Nashville, Tennessee

## Abstract

**Importance:**

Inguinal hernia repair (IHR) is one of the most commonly performed operations in the United States, with approximately 800 000 performed each year. US veterans are a unique group that is predominantly male and tends to have higher rates of medical comorbidities and tobacco use, common risk factors for hernia development. With significant advancements in IHR technique in recent years, the majority of national database studies have focused on civilian populations.

**Objective:**

To examine trends and the evolution of IHR within the Veterans Health Administration (VHA) over the past 20 years.

**Design, Setting, and Participants:**

This cohort study examined national data retrospectively from all VHA institutions in the period 2002 to 2022. Participants were veterans who received IHR within the VHA were identified through the Corporate Data Warehouse using procedure and diagnosis codes. Data were analyzed from January to April 2025.

**Exposure:**

Inguinal hernia repair.

**Main Outcomes and Measures:**

Surgical volumes and trends in IHR surgical approach across the VHA were examined over time. Additional measures included procedural urgency and surgical setting.

**Results:**

A total of 308 876 veterans underwent IHR from 2002 to 2022. Patients were predominantly male (306 905 [99.4%]; 1971 female patients [0.6%]), with a median (IQR) age of 65 (57-73) years. They had high rates of hypertension (198 792 [64.4%]), depression (102 880 [33.3%]), chronic obstructive pulmonary disease (82 608 [26.7%]) and alcohol use disorder (68 427 [22.2%]). A total of 60 088 patients (19.5%) underwent minimally invasive (MIS) IHR while 246 403 procedures (79.8%) were open. Most were outpatient (300 400 [97.3%]); only 17 352 (5.6%) were urgent or emergent. Compared with men, women were more likely to undergo MIS-IHR (452 women [22.9%] vs 59 636 men [19.4%]) and urgent/emergent procedures (218 women [11.1%] vs 17 134 men [5.6%]). Annual IHR volume increased from 12 086 in 2002 to 17 897 in 2022, with an increase in MIS-IHR from 1102 (9.2%) to 7286 (41.0%). After a decline during COVID-19, IHR numbers have since recovered and surpassed pre-2020 volume. Throughout the study period, IHRs performed in the community were less likely to be urgent or emergent cases (1006 [3.0%] vs 16 346 [5.9%] in Veterans Affairs [VA] facilities).

**Conclusions and Relevance:**

This study found that over the past 20 years, the volume of IHRs performed in the VHA system has increased, along with MIS approaches. Hernia repairs are a significant surgical burden within the veteran population, reflecting the evolving surgical practices and needs of this unique demographic.

## Key Points

**Question:**

How have volume and surgical approach to inguinal hernia repair (IHR) in veterans changed within the Veterans Health Administration (VHA) over the last 20 years?

**Findings:**

This cohort study involving 308 876 veterans (2002 to 2022) found that IHR volume increased nearly 50%, with minimally invasive approaches increasing from 9% to 41%. Community-based procedures were less often urgent/emergent, and female veterans had higher rates of minimally invasive surgery and urgent repairs.

**Meaning:**

These results highlight significant growth in IHR volume and a shift toward minimally invasive approaches in the VHA, illustrating evolving technical practice and veterans’ surgical needs, providing data to inform policy and resource allocation.

## Teaser

This cohort study examines 20 years of data for US veterans who had inguinal hernia repair to track trends and changes in volume and surgical approach.

## Introduction

Hernia repair is one of the most commonly performed general surgery procedures worldwide. In the US, approximately 800 000 inguinal or femoral hernia repairs are conducted annually.^1^ The lifetime risk of inguinal hernia is estimated at 27% to 43% for men and 3% to 6% for women.^2^ Abdominal core disease, which includes both ventral and groin hernias, can lead to both acute and chronic health issues, significantly affecting quality of life.^3^ Management is multidisciplinary and may include surgery, medical therapy, physical rehabilitation, and preventive strategies.^3^ From a public health standpoint, abdominal core health represents a substantial financial burden, with inguinal hernia repair (IHR) accounting for nearly $2.5 billion in annual health care costs in the early 2000s.^1^

Over the past few decades, surgical techniques for IHR have evolved. Various populations undergoing IHR have been studied to generate comparative data on the relative effectiveness of surgical approaches. While initial randomized clinical trial (RCT) results found watchful waiting to be an acceptable approach for men with minimally symptomatic IHR, long-term follow-up has demonstrated that most patients (68.0%) ultimately require surgery because of the progression of symptoms.^4,5^ An RCT from 14 Veterans Affairs Medical Centers (VAMCs) published in 2004 concluded that open IHR was superior to minimally invasive surgery (MIS) for IHR in terms of recurrence and complications in a primarily male population, although MIS was associated with faster recovery.^6^ A follow-up cost-effectiveness study reported higher costs for MIS with no improvement in quality-adjusted life-years.^7^ In contrast, recent civilian studies have demonstrated favorable outcomes after MIS-IHR when compared with open IHR, including less pain; earlier return to work; equivalent postoperative mortality, morbidity, and readmission rates; and lower recurrence rates.^8,9,10^

Despite these advances, little is known about contemporary trends in overall IHR case volume and surgical approach within the Veterans Health Administration (VHA). Most national studies reflect civilian populations, and while the Veterans Affairs Surgical Quality Improvement Program (VASQIP) has examined IHR outcomes by technique, the database does not capture the full scope of IHR volume or care settings across the VHA.^1,11,12,13,14^

Veterans represent a distinct population from civilian patients: typically older, predominantly male, and with higher rates of comorbidities such as obesity, smoking, and diabetes.^9,10,11,12,13,14^ Understanding IHR trends within this group is essential to improving care delivery, informing resource allocation, and guiding public health efforts. This study aims to characterize national trends in IHR within the VHA over the past 2 decades, including procedure volume, approach, urgency, and care setting (VAMC facilities and non-VA community care settings).

## Methods

We queried the VA Corporate Data Warehouse (CDW), a national repository of electronic health records for all VAMC facilities, to select all veterans who have undergone IHR from 2002 to 2022. Veterans older than 18 years who underwent open or MIS-IHR were identified based on *Current Procedural Terminology* (*CPT*) codes and administrative codes from the *International Classification of Diseases, Ninth Revision, Clinical Modification* (*ICD-9-CM*), and *International Statistical Classification of Diseases, Tenth Revision, Clinical Modification* (*ICD-10-CM*). A crosswalk of all codes used for data acquisition is included in eTable 1 in Supplement 1. As minimally invasive vs open technique is not captured in IHR *ICD-9-CM* codes, veterans with only *ICD-9-CM* codes identifying their procedure (without any corresponding *CPT* codes) were excluded from analyses of surgical approach (2385 patients, 0.77% of the cohort). MIS-IHR included both laparoscopic and robotic hernia repairs. This study was approved by the Tennessee Valley Healthcare System Veteran Affairs Medical Center institutional review board and research and development committees (VA IRBNet No. 321909), with approval for waived informed consent. This study is reported in accordance with the Strengthening the Reporting of Observational Studies in Epidemiology (STROBE) reporting guidelines.

Preoperative demographic details and patient characteristics obtained from the CDW for each veteran included age, sex, self-reported race, and comorbidities. The Elixhauser comorbidity score was used as a composite validated weighted index of comorbidities based on *ICD-9-CM/ICD-10-CM* diagnosis codes.^15^ Details on select comorbidities were obtained, including hypertension, diabetes, human immunodeficiency virus status, congestive heart failure (CHF), chronic obstructive pulmonary disease (COPD), depression, and alcohol and drug use disorders. Operative characteristics encompass geographic region, urgency (elective vs urgent/emergent), inpatient vs outpatient setting, and location of service delivery (within a VAMC facility or through community care). Community care procedures include those that were performed outside of VAMC facilities but reimbursed by the VHA, classified using Fee Basis records. Data were incomplete for 48 004 Elixhauser comorbidity scores (15.5%) and 183 surgical urgency classifications (0.06%). Demographic and operative characteristics are represented by frequencies and percentages for categorial variables and median (IQR) for continuous variables.

Primary outcomes included the incidence and trends of open and minimally invasive hernia repairs performed in the inpatient and outpatient settings throughout the study period. We examined patient demographics by procedure type. Demographic characteristics, changes in surgical approach, urgency, and care setting were detailed over time. We report both the absolute number of groin hernia repairs and the IHR rate standardized to the annual population of veterans receiving VHA care, defined as the number of unique individuals with at least 1 VHA visit per year. Additional analyses compared patient and operative characteristics by sex and by site of care (VAMC facility vs community care). To explore changes in VAMC facility vs community care IHR volume and procedure type, we also performed comparisons of procedures performed before and after the passage of the MISSION Act of 2018, which expanded access to community-based care.^16^

χ^2^ Tests were used to compare categorical variables, and Wilcoxon rank sum tests were applied to continuous variables for nonparametric distributions. All data analysis was performed using R Studio version 2023.12.1 + 402 (R Foundation) during the period January to April 2025.

## Results

From 2002 to 2022, a total of 308 876 veterans underwent IHR performed through the VHA. On average, throughout the study period, more than 14 700 IHRs of any kind were performed each year. Of these, 60 088 (19.5%) were minimally invasive while 246 403 (79.8%) were open surgeries, with 2385 (0.8%) unknown because of *ICD-9-CM* coding alone. Among MIS procedures, 49 802 (82.9%) were laparoscopic and 10 286 (17.1%) robotic. Most of these operations were performed on an elective basis (291 341 [94.4%]), while 17 352 (5.6%) took place in urgent or emergent settings. IHRs were predominantly outpatient procedures (300 400 [97.3%]) vs inpatient procedures (8476 [2.7%]). The median (IQR) age of the cohort was 65 (57-73) years, and the majority of veterans were male (306 905 [99.4%] vs 1971 female veterans [0.6%]) and White (238 358 [77.2%] compared with 1761 [0.6%] American Indian or Alaska Native, 915 [0.3%] Asian, 45 226 [14.6%] Black or African American, and 1885 [0.6%] Native Hawaiian or Other Pacific Islander veterans and 20 731 [6.7%] with unknown race). Veterans had high rates of hypertension (198 792 [64.4%]), depression (102 880 [33.3%]), COPD (82 608 [26.7%]), and alcohol use disorder (68 427 [22.2%]). The highest proportion of IHR were performed in the South (126 059 [40.8%]), followed by the West (73 629 [23.8%]) and Midwest (66 930 [21.7%]).

Demographic characteristics for veterans who underwent MIS vs open IHR in the inpatient vs outpatient settings are detailed in Table 1. Patients who underwent inpatient IHR tended to be older (median [IQR] age, 69 [62-78] years vs 65 [57-73] years; *P* < .001) and have higher Elixhauser comorbidity scores (median [IQR] score, 8 [−2 to 22] vs 1 [−4 to 10]; *P* < .001). The most common procedure performed was the outpatient open IHR (241 241 [78.7%]), followed by MIS outpatient surgery (59 134 [19.3%]). In the urgent/emergent setting, only 1787 IHRs (10.0%) were performed MIS vs 58 234 elective procedures (20.0%). Veterans requiring urgent/emergent IHR were more likely to have comorbidities, including diabetes (4420 [25.5%] vs 55 933 [19.2%]; *P* < .001), CHF (3085 [17.8%] vs 23 584 [8.1%]; *P* < .001), and COPD (5874 [33.8%] vs 76 690 [26.3%]; *P* < .001). Most IHRs performed at VAMC facilities were open outpatient (222 477 [80.7%]) or MIS outpatient (47 150 [17.1%]), and most IHRs through community care were MIS outpatient (11 984 [36.1%]) or open outpatient (18 764 [56.5%]) procedures.

**Table 1.** Clinical and Demographic Characteristics of Patients Undergoing IHR

Characteristic	Outpatient (n = 300 375), No. (%)		Inpatient (n = 6116), No. (%)	
	MIS (n = 59 134)	Open (n = 241 241)	MIS (n = 954)	Open (n = 5162)
Age, median (IQR), y	64 (55 to 71)	65 (57 to 73)	68 (61 to 75)	71 (63 to 79)
Gender				
Female	426 (0.7)	1407 (0.6)	26 (2.7)	72 (1.4)
Male	58 708 (99.3)	239 834 (99.4)	928 (97.3)	5090 (98.6)
Race^a^				
American Indian or Alaska Native	417 (0.7)	1299 (0.5)	5 (0.5)	28 (0.5)
Asian	206 (0.3)	678 (0.3)	4 (0.4)	15 (0.3)
Black or African American	7537 (12.7)	36 158 (14.9)	147 (15.4)	967 (18.7)
Native Hawaiian or Other Pacific Islander	315 (0.5)	1500 (0.6)	6 (0.6)	38 (0.7)
White	47 071 (79.6)	185 043 (76.7)	749 (78.5)	3800 (73.6)
Unknown	3588 (6.1)	16 563 (6.9)	43 (4.5)	314 (6.1)
Elixhauser comorbidity score, median (IQR)	0 (−5 to 8)	1 (−2 to 10)	6 (−2 to 17)	10 (0 to 24)
Selected comorbidities				
Hypertension	35 941 (60.8)	156 298 (64.8)	713 (74.7)	4125 (79.9)
Diabetes	10 992 (18.6)	46 902 (19.4)	275 (28.8)	1631 (31.6)
HIV	536 (0.9)	2051 (0.9)	9 (0.9)	44 (0.9)
CHF	3515 (5.9)	21 336 (8.8)	146 (15.3)	1249 (24.2)
COPD	13 235 (22.4)	66 001 (27.4)	316 (33.1)	2104 (40.8)
Depression	22 362 (37.8)	77 143 (32.0)	414 (43.4)	2157 (41.8)
Alcohol use disorder	14 026 (23.7)	52 082 (21.6)	247 (25.9)	1485 (28.8)
Drug use disorder	14 602 (24.7)	45 103 (18.7)	276 (28.9)	1508 (29.2)
Region				
Midwest	13 925 (23.5)	51 192 (21.2)	246 (25.8)	1114 (21.6)
Northeast	6245 (10.5)	30 392 (12.6)	130 (13.6)	786 (15.2)
South	23 898 (40.4)	98 973 (41.0)	325 (34.1)	1855 (35.9)
Unknown	3 (<0.1)	4 (<0.1)	0	1 (<0.1)
US outlying islands	471 (0.8)	3708 (1.5)	49 (5.1)	111 (2.2)
West	14 592 (24.7)	56 972 (23.6)	204 (21.4)	1295 (25.1)
Urgency				
Elective	57 538 (97.3)	228 064 (94.5)	696 (73.0)	2873 (55.7)
Urgent/emergent	1530 (2.6)	13 068 (5.4)	257 (26.9)	2282 (44.2)
Setting				
VAMC facility	47 150 (79.7)	222 477 (92.2)	810 (84.9)	4224 (81.8)
Community care	11 984 (20.3)	18 764 (7.8)	144 (15.1)	938 (18.2)

Abbreviations: CHF, congestive heart failure; COPD, chronic obstructive pulmonary disease; HIV, human immunodeficiency virus; IHR, inguinal hernia repair; MIS, minimally invasive surgery; VAMC, Veterans Affairs Medical Center.

^a^ Race data were obtained from the VA Corporate Data Warehouse and self-reported by the participants.

Patient and operative characteristics were stratified by sex (Table 2). Female veterans were more likely to undergo IHR on an inpatient basis (138 [7.0%] vs 8338 male veterans [2.7%]) and more likely to undergo surgery in the community (309 [15.7%] vs 32 904 male veterans [10.7%]). Women undergoing IHR had nearly double the incidence of requiring urgent/emergent intervention (218 [11.1%] vs 17 134 male veterans [5.6%]; *P* < .001). Women were slightly more likely to undergo MIS-IHR than men (452 [22.9%] vs 59 636 [19.4%], respectively; *P* < .001), including both laparoscopic (348 [17.7%] vs 49 454 [16.1%], respectively) and robotic (104 [5.4%] vs 10 182 [3.3%], respectively) approaches. Compared with their male counterparts, women veterans who underwent IHR were younger (median [IQR] age, 56 [44-65] vs 65 [57-73] years for men), and less likely to have hypertension (917 [46.5%] vs 197 875 men [64.5%]), diabetes (257 [13.0%] vs 60 137 men [19.6%]), CHF (93 [4.7%] vs 26 603 men [8.7%]), and more likely to have depression (1097 [55.7%] vs 101 783 men [33.2%]).

**Table 2.** Inguinal Hernia Repairs in Female and Male Veterans

Characteristic	No. (%)		*P* value
	Female (n = 1971)	Male (n = 306 905)	
Age, median (IQR), y	56 (44 to 65)	65 (57 to 73)	<.001
Race^a^			
American Indian or Alaska Native	22 (1.1)	1739 (0.6)	<.001
Asian	12 (0.6)	903 (0.3)	
Black or African American	365 (18.5)	44 861 (14.6)	
Native Hawaiian or Other Pacific Islander	13 (0.7)	1872 (0.6)	
White	1421 (72)	236 937 (77.2)	
Unknown	138 (7.0)	20 593 (6.7)	
Elixhauser comorbidity score, median (IQR)	−2 (−5 to 7)	1 (−3 to 10)	<.001
Selected comorbidities			
Hypertension	917 (46.5)	197 875 (64.5)	<.001
Diabetes	257 (13.0)	60 137 (19.6)	<.001
HIV	11 (0.6)	2654 (0.9)	.14
CHF	93 (4.7)	26 603 (8.7)	<.001
COPD	555 (28.2)	82 053 (26.7)	.20
Depression	1097 (55.7)	101 783 (33.2)	<.001
Alcohol use disorder	322 (16.3)	68 105 (22.2)	<.001
Drug use disorder	399 (20.2)	61 485 (20.0)	.80
Region			
Midwest	356 (18.1)	66 574 (21.7)	<.001
Northeast	219 (11.1)	37 661 (12.3)	
South	853 (43.3)	125 206 (40.8)	
Unknown	1 (<0.1)	7 (<0.1)	
US outlying islands	10 (0.5)	4360 (1.4)	
West	532 (27.0)	73 097 (23.8)	
Hospitalization			
Outpatient procedure	1833 (93.0)	298 567 (97.3)	<.001
Inpatient procedure	138 (7.0)	8338 (2.7)	
Procedure type			
Minimally invasive	452 (22.9)	59 636 (19.4)	<.001
Open	1479 (75.0)	244 924 (79.8)	
Urgency			
Elective	1751 (88.8)	289 590 (94.3)	<.001
Urgent/emergent	218 (11.1)	17 134 (5.6)	
Setting			
Community care	309 (15.7)	32 904 (10.7)	<.001
VAMC facility	1662 (84.3)	274 001 (89.3)	

Abbreviations: CHF, congestive heart failure; COPD, chronic obstructive pulmonary disease; HIV, human immunodeficiency virus; MIS, minimally invasive surgery; VAMC, Veterans Affairs Medical Center.

^a^ Race data were obtained from the VA Corporate Data Warehouse and self-reported by the participants.

**Figure 1.**
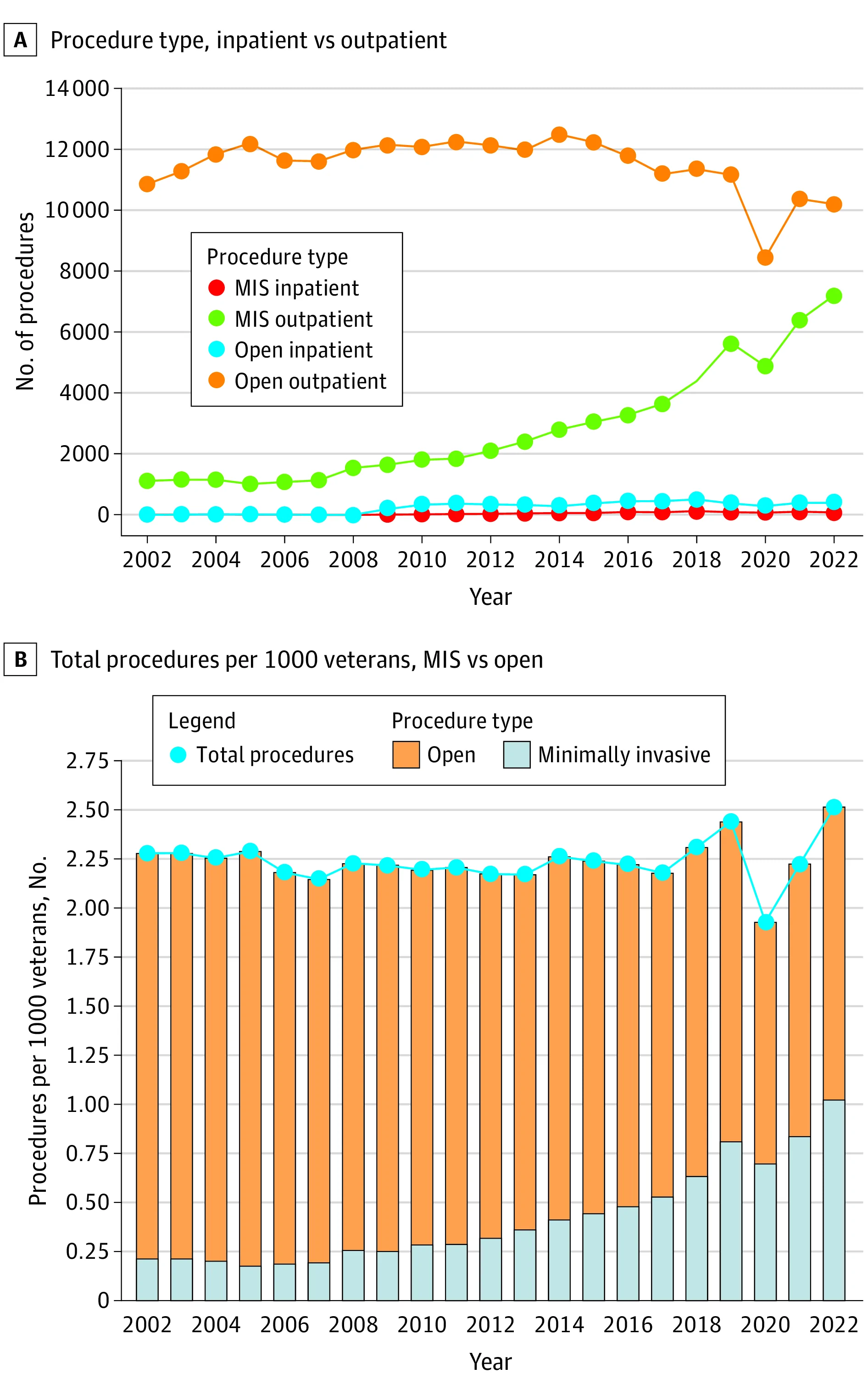
Dot Plot and Bar Graph Showing Inguinal Hernia Repair Procedure Type for US Veterans, 2002-2022 A, Total number of minimally invasive surgery (MIS) vs open groin hernia repair in the inpatient and outpatient settings over 20 years. B, Total number of groin hernia repairs per 1000 veterans receiving care through the Veterans Health Association per year.

Throughout the study period, overall IHR volume increased from 12 086 IHR in 2002 to 17 897 in 2022, yielding an increase of approximately 48.1% throughout the study period. Figure 1A demonstrates the annual trends in IHR technique and admission status over 21 years. Use of MIS-IHR increased substantially from 1102 (9.2%) in 2002 to 7286 (41.0%) in 2022. Use of robotic IHR techniques also increased from 9 (<0.1%) in 2007 to 2781 (16%) in 2022. When adjusted for the total number of veterans receiving VHA care per year, the number of IHRs ultimately increased incrementally from 2.28 IHRs per 1000 veterans in 2002 to 2.51 in 2022 (Figure 1B).

**Figure 2.**
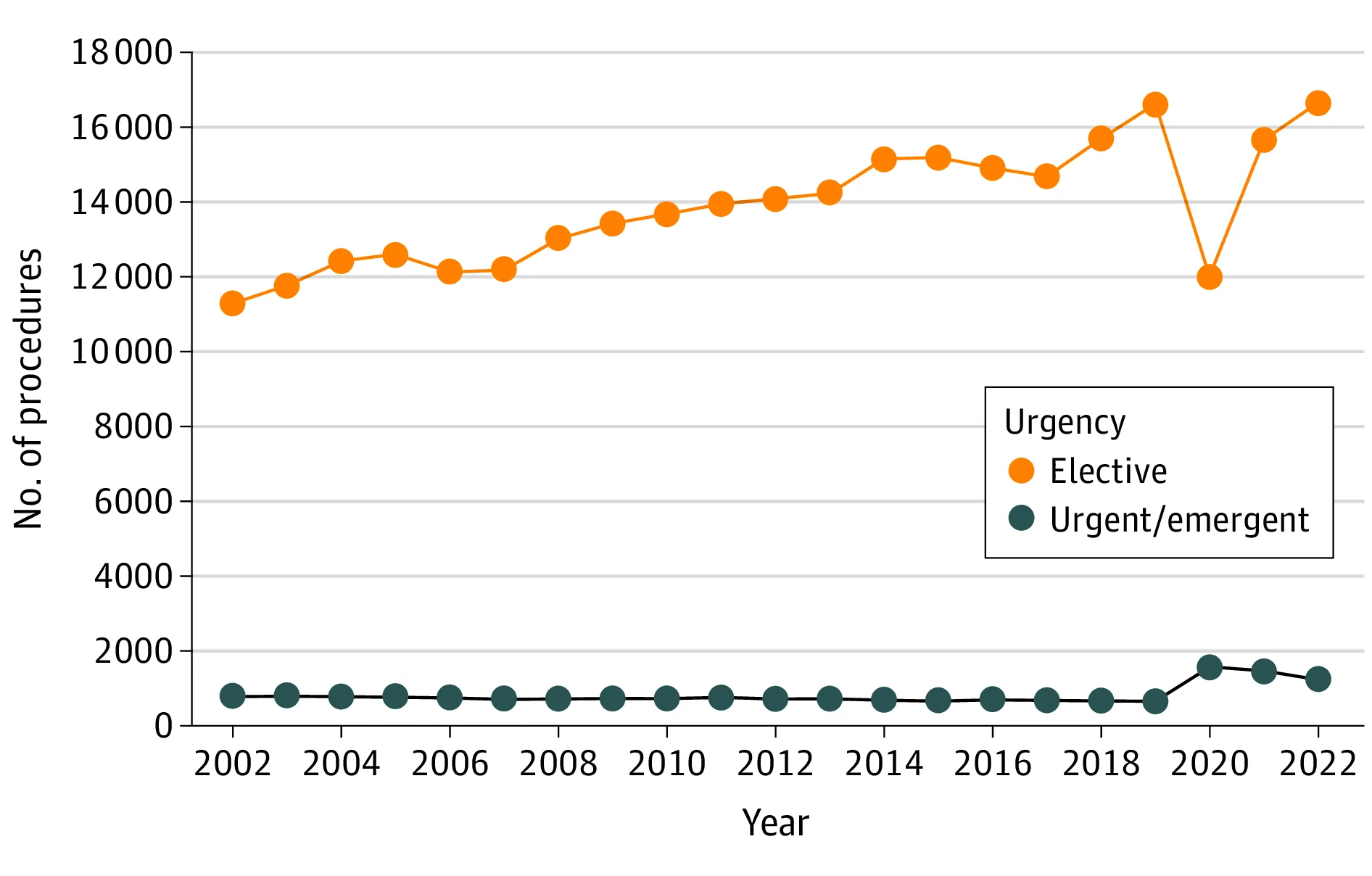
Dot Plot Showing Urgency of Inguinal Hernia Repairs for US Veterans, 2002-2022

Procedure volume temporarily declined during the COVID-19 pandemic, with the most pronounced decrease seen in open repairs (Figure 1A). As shown in Figure 2, the proportion of urgent or emergent cases increased from 13 061 (5.0%) prepandemic to 4291 (8.8%) from 2020 to 2022. IHR volumes rebounded after 2020, increasing from 1.93 per 1000 veterans in 2020 to 2.51 in 2022, surpassing the prepandemic rate of 2.43 in 2019 (Figure 1B).

After the implementation of expanded community care access in 2018, the proportion of procedures performed outside VAMC facilities more than quadrupled (11 512 [5.1%] to 21 701 [26.3%]). Differences in patient and operative characteristics between VAMC facilities and community-based surgeries are outlined in Table 3. Veterans treated in the community were older (median [IQR] age, 68 [59-74] vs 65 [57-72] years for the VAMC-treated group; *P* < .001), more likely to be female (309 [0.9%] vs 1662 [0.6%] for the VAMC-treated group; *P* < .001), and had greater comorbidity burdens (median [IQR] Elixhauser score, 3 [−2 to 14] vs 1 [−3 to 10] for the VAMC-treated group; *P* < .001), particularly with higher rates of diabetes (7920 [23.8%] for community care vs 52 474 [19.0%] for the VAMC-treated group) and CHF (3946 [11.9%] vs 22 750 [8.3%] for the VAMC-treated group). Community-based IHRs were less often performed urgently or emergently (1006 [3.0%] vs 16 346 [5.9%] for the VAMC-treated group; *P* < .001). When comparing community care vs VAMC IHR by procedure type, 19 702 (59.3%) vs 226 701 (82.2%) were open; 10 715 (32.3%) vs 39 087 (14.2%) laparoscopic; and 1413 (4.3%) vs 8873 (3.2%) robotic, respectively.

**Table 3.** Inguinal Hernia Repair by VAMC vs Community Care Setting (n = 308 876)

Characteristic	Community care (n = 33 213)	VAMC (n = 275 663)	*P* value
Age, median (IQR), y	68 (59 to 74)	65 (57 to 72)	<.001
Gender			
Female	309 (0.9)	1662 (0.6)	<.001
Male	32 904 (99.1)	274 001 (99.4)	
Race^a^			
American Indian or Alaska Native	270 (0.8)	1491 (0.5)	<.001
Asian	136 (0.4)	779 (0.3)	
Black or African American	3135 (9.4)	42 091 (15.3)	
Native Hawaiian or Other Pacific Islander	313 (0.9)	1572 (0.6)	
White	26 917 (81.0)	211 441 (76.7)	
Unknown	2442 (7.4)	18 289 (6.6)	
Elixhauser comorbidity score, median (IQR)	3 (−2 to 14)	1 (−3 to 10)	<.001
Selected comorbidities			
Hypertension	22 944 (69.1)	175 848 (63.8)	<.001
Diabetes	7920 (23.8)	52 474 (19.0)	<.001
HIV	167 (0.5)	2498 (0.9)	<.001
CHF	3946 (11.9)	22 750 (8.3)	<.001
COPD	9296 (28.0)	73 312 (26.6)	<.001
Depression	13 230 (39.8)	89 650 (32.5)	<.001
Alcohol use disorder	8267 (24.9)	60 160 (21.8)	<.001
Drug use disorder	9746 (29.3)	52 138 (18.9)	<.001
Region			
Midwest	8689 (26.2)	58 241 (21.1)	<.001
Northeast	2641 (8.0)	35 239 (12.8)	
South	12 092 (36.4)	113 966 (41.3)	
Unknown	8 (0.02)	0	
US outlying islands	138 (0.4)	4232 (1.5)	
West	9645 (29.0)	63 985 (23.2)	
Inpatient vs outpatient			
Outpatient	30 749 (92.6)	269 651 (97.8)	<.001
Inpatient	2464 (7.4)	6012 (2.2)	
Procedure type			
Minimally invasive	12 128 (36.5)	47 960 (17.4)	<.001
Open	19 702 (59.3)	226 701 (82.2)	
Urgency			
Elective	32 025 (96.4)	259 316 (94.1)	<.001
Urgent/emergent	1006 (3.0)	16 346 (5.9)	

Abbreviations: CHF, congestive heart failure; COPD, chronic obstructive pulmonary disease; HIV, human immunodeficiency virus; VAMC, Veterans Affairs Medical Center.

^a^ Race data were obtained from the VA Corporate Data Warehouse and self-reported by the participants.

## Discussion

This study offers a contemporary overview of IHR practice patterns across the VHA. Over the past 2 decades, IHR volume increased by 48.1%, with a 4.5-fold increase in MIS-IHR. Emergency repairs were more common among women and in community care. IHR represents a substantial and growing source of health care utilization for veterans.

While IHR trends are well documented in civilian populations, data for veterans remain limited. In England, inguinal hernias comprise approximately 71% of abdominal wall hernia repairs, with men facing a 9-fold higher lifetime risk.^2,17^ US estimates reported about 800 000 groin hernia repairs in 2003 and an 18.4% increase in groin and abdominal hernia burden from 1990 to 2019.^1,18^ Although our study covers a more contemporary time period and is specific to IHR, growth within the VHA exceeds this national trend, increasing 48.1% over 21 years.

Early VA Hernia Cooperative Study trials favored open repair based on recurrence, complications, and cost, though subsequent analyses suggested surgeon experience in MIS and older age may have contributed to these findings.^6,7,19^ Another RCT including 2 VAMCs initially supported watchful waiting for minimally symptomatic IHR, with a hernia accident rate of 0.0018 events per patient-year. However, long-term follow-up demonstrated that 68.0% of patients ultimately required surgery because of symptom progession.^4,5^ Our data reflect these shifts: MIS usage initially declined after the 2004 RCT but then rose steadily, even after the 2006 cost-effectiveness study favoring open repair. Similarly, total IHR volume dipped after the 2006 watchful-waiting RCT but rebounded following the 2013 long-term results. This suggests that VA-specific evidence does influence VA practice, but broader shifts in surgical innovation and training also play a role.

MIS adoption in the VHA has rapidly expanded despite earlier studies supporting open techniques. According to the Americas Hernia Society Quality Collaborative, MIS-IHR increased from 14% in 2003 to 58% in 2019.^1,12^ A National Surgical Quality Improvement Program (NSQIP) study from 2006 to 2017 demonstrated increase in MIS-IHR from 23.1 to 37.8%.^13^ Our data show a parallel rise of MIS-IHR within the VHA, from 9.2% in 2002 to 41.0% in 2022, as well as an increase in robotic IHR from 0.1% in 2007 to 16% in 2022, reflecting broader national trends in the VA and US.^20^ While the majority of IHRs in the VHA remain open, a decline from 91% to 59% in 2022 demonstrates substantial MIS adoption and improving alignment with contemporary guideline recommendations, likely reflecting increased MIS training, evolving surgeon experience, and broader dissemination of MIS techniques and resources within the VA. Prior VA analyses using VASQIP data reported similar proportions of MIS use, though VASQIP does not reflect total procedural volume, as our study does.^14^ Continued efforts to expand MIS approaches in VA practice are likely to further benefit eligible patients.

Our findings reveal notable differences between VA and civilian populations. In Medicare data, women comprised 13.6% of groin hernia repairs, while women made up just 0.6% of our IHR cohort, reflecting national veteran demographics.^21,22^ Interestingly, women in the Medicare study had a higher prevalence of comorbidities compared with men and were less likely to undergo MIS repairs than men, while our female veterans had a decreased comorbidity burden and were more likely to undergo MIS-IHR.^21^ Women veterans have more than double the risk of requiring an emergency hernia repair. These differences may arise because of a lower average age with associated lower comorbidity profiles, differential practice patterns, missed diagnoses in women, access to equipment or operative time, surgeon or patient preferences, intrinsic differences in pelvic anatomy, or other unidentified reasons. However, it should be noted that the increased use of MIS-IHR for women through the VA is in accordance with current international guidelines.^23^

Furthermore, urgency trends diverged between the VA and civilian populations. In the National Center for Health Statistics National Hospital Discharge Survey, there was an increase in emergent groin hernia repairs from 6.1 per 100 000 person-years in 2001 to 6.4 per 100 000 person-years in 2010.^24^ In our cohort, 6.5% of veterans underwent urgent/emergent repairs in 2002, which decreased to 5.0% in 2010.

Our data reflect the significant drop in elective surgical volume and increase in urgent/emergent IHR during the COVID-19 pandemic, aligning with national directives that resulted in a 75.0% reduction in elective procedures at the VA in 2020.^25^ When compared with total veteran encounters, elective cases decreased from 2.35 per 1000 encounters in 2019 to 1.70 in 2020 and urgent/emergent encounters increased from 0.09 to 0.23. While a similar decrease in elective hernia repairs (around 21.0%) during the pandemic was seen in a population analysis in Canada, Gomez et al^26^ did not see a difference in the rate of urgent IHR performed during this time. The cause of this increase in total urgent/emergent cases could be associated with recategorization of highly symptomatic hernias from “elective” to “urgent/emergent” status to overcome barriers to care. Ultimately, IHR volume through the VHA recovered and surpassed prepandemic levels, from 13 630 IHRs in 2020 to 17 897 in 2022.

Veterans are known to be older and carry a higher burden of comorbidities than the general population, which may contribute to increased hernia-related health care utilization.^27,28,29,30,31,32^ In our cohort, medical comorbidities were notably more common than previously reported in Medicare populations of patients undergoing IHR.^21^

The VHA is the nation’s only integrated, federally operated single-payer health care system. Maintaining surgical innovation is key to delivering high-quality care. Policies like the Veterans Choice Act (2014) and MISSION Act (2018) have expanded access to community-based care, increasing the number of procedures performed outside of VAMC facilities.^16,33^ Various studies have found VAMC outcomes to be comparable or better than community hospitals.^34,35,36,37^ While Graham et al^34^ found that veterans referred to non-VA care were typically younger and White with fewer comorbidities, our findings reveal that community-based IHRs tended to involve more medically complex patients. This may reflect referrals of complex cases to academic centers or triage decisions for treatment at closest geographic facilities. Nevertheless, the majority of elective IHRs among veterans are performed at VAMC facilities, a trend consistent with prior research demonstrating veterans’ preferences to remain in the VA system.^38,39^

Understanding shifts in surgical practice is vital given implications for cost and quality. In 2003, repair of abdominal wall hernias cost nearly $2.5 billion annually.^1^ More recent estimates according to NSQIP in 2016 placed the average cost at $4360 for open and $5105 for MIS-IHR repairs.^40^ Applying these costs to our observed volumes over this 20-year period, the projected cost of veteran IHRs would exceed $1.38 billion if delivered in the private sector. These figures highlight the importance of improving hernia prevention and reducing recurrence, both to contain costs and enhance quality of care.

Although this study did not directly evaluate prevention or recurrence strategies, the high IHR burden observed underscores the importance of actionable opportunities to improve quality and reduce costs. Evidence-based interventions, including smoking cessation, weight optimization, glycemic control, risk stratification, and prehabilitation, reduce complications and recurrence and can integrate into existing VHA preoperative pathways.^41,42,43,44,45,46,47,48,49,50,51^ Guideline-concordant use of MIS techniques and resources may further improve outcomes.^52,53,54,55^ Postoperative, structured follow-up and patient education (leveraging the VHA’s robust telehealth infrastructure) offers scalable approaches to early complication detection and recurrence mitigation.^47,56,57,58^ The VHA’s integrated data systems support longitudinal outcome tracking and continuous quality improvement.^36,59,60^ eTable 2 in Supplement 1 summarizes evidence-supported interventions and potential implementation pathways within the VA.

### Limitations

Limitations of this study include potential data inaccuracies inherent to large database analyses. For veterans who received care at non-VAMC facilities funded by the VHA, missing or inconsistent data may arise due to differences in electronic medical records. For consistency, comorbidity data were gathered from the CDW, and therefore, there may be additional missing data regarding comorbidities diagnosed in patients through community care facilities. Key clinical details, such as patient outcomes, recurrence rates, wound classification, hernia size and location, mesh use, and cost, were not captured and remain important areas for future investigation.

## Conclusions

This study found that over the past 20 years, the volume of IHRs performed in the VHA system has increased, along with MIS approaches. IHRs continue to represent a substantial part of the surgical workload in the veteran population, highlighting both changing surgical practices and the distinct health care needs of this group. Our findings reflect parallel growth and innovation within the VHA compared with civilian systems but also underscore the broader public health significance of abdominal core health for both veterans and civilians. Increased investment is needed to enhance care quality, support research, track treatment outcomes, and prioritize hernia prevention.

## Notes

Supplement 1. **eTable 1. **Crosswalk of CPT and ICD codes for inguinal hernia repairs by category **eTable 2.** Evidence-Based Interventions to Improve Quality and Reduce Recurrence After Inguinal Hernia Repair: Potential Applications Within the Veterans Health Administration

Supplement 2. Data sharing statement

## References

[1] Rutkow IM. Demographic and socioeconomic aspects of hernia repair in the United States in 2003. Surg Clin North Am. 2003;83(5):1045-1051, v-vi. doi:10.1016/S0039-6109(03)00132-4 14533902

[2] Primatesta P, Goldacre MJ. Inguinal hernia repair: incidence of elective and emergency surgery, readmission and mortality. Int J Epidemiol. 1996;25(4):835-839. doi:10.1093/ije/25.4.835 8921464

[3] Poulose BK, Adrales GL, Janis JE. Abdominal core health: a needed field in surgery. JAMA Surg. 2020;155(3):185-186. doi:10.1001/jamasurg.2019.5055 31851303

[4] Fitzgibbons RJ Jr, Giobbie-Hurder A, Gibbs JO, . Watchful waiting vs repair of inguinal hernia in minimally symptomatic men: a randomized clinical trial. JAMA. 2006;295(3):285-292. doi:10.1001/jama.295.3.285 16418463

[5] Fitzgibbons RJ Jr, Ramanan B, Arya S, ; Investigators of the Original Trial. Long-term results of a randomized controlled trial of a nonoperative strategy (watchful waiting) for men with minimally symptomatic inguinal hernias. Ann Surg. 2013;258(3):508-515. doi:10.1097/SLA.0b013e3182a19725 24022443

[6] Neumayer L, Giobbie-Hurder A, Jonasson O, ; Veterans Affairs Cooperative Studies Program 456 Investigators. Open mesh versus laparoscopic mesh repair of inguinal hernia. N Engl J Med. 2004;350(18):1819-1827. doi:10.1056/NEJMoa040093 15107485

[7] Hynes DM, Stroupe KT, Luo P, . Cost effectiveness of laparoscopic versus open mesh hernia operation: results of a Department of Veterans Affairs randomized clinical trial. J Am Coll Surg. 2006;203(4):447-457. doi:10.1016/j.jamcollsurg.2006.05.019 17000387

[8] Wauschkuhn CA, Schwarz J, Boekeler U, Bittner R. Laparoscopic inguinal hernia repair: gold standard in bilateral hernia repair? results of more than 2800 patients in comparison to literature. Surg Endosc. 2010;24(12):3026-3030. doi:10.1007/s00464-010-1079-x 20454807

[9] Charles EJ, Mehaffey JH, Tache-Leon CA, Hallowell PT, Sawyer RG, Yang Z. Inguinal hernia repair: is there a benefit to using the robot? Surg Endosc. 2018;32(4):2131-2136. doi:10.1007/s00464-017-5911-4 29067575 PMC10740385

[10] Liem MSL, van Duyn EB, van der Graaf Y, van Vroonhoven TJMV; Coala Trial Group. Recurrences after conventional anterior and laparoscopic inguinal hernia repair: a randomized comparison. Ann Surg. 2003;237(1):136-141. doi:10.1097/00000658-200301000-00019 12496541 PMC1513978

[11] Rogers AP, Xu Y, Lidor AO. Healthcare resource utilization in inguinal hernia repair: a three-year cost evaluation of Truven Health Marketscan research databases. J Surg Res. 2021;264:408-417. doi:10.1016/j.jss.2021.02.041 33848840

[12] AlMarzooqi R, Tish S, Huang LC, Prabhu A, Rosen M. Review of inguinal hernia repair techniques within the Americas Hernia Society Quality Collaborative. Hernia. 2019;23(3):429-438. doi:10.1007/s10029-019-01968-y 31069581

[13] Madion M, Goldblatt MI, Gould JC, Higgins RM. Ten-year trends in minimally invasive hernia repair: a NSQIP database review. Surg Endosc. 2021;35(12):7200-7208. doi:10.1007/s00464-020-08217-9 33398576

[14] Holleran TJ, Napolitano MA, Sparks AD, Duncan JE, Garrett M, Brody FJ. Trends and outcomes of open, laparoscopic, and robotic inguinal hernia repair in the veterans affairs system. Hernia. 2022;26(3):889-899. doi:10.1007/s10029-021-02419-3 33909151

[15] van Walraven C, Austin PC, Jennings A, Quan H, Forster AJ. A modification of the Elixhauser comorbidity measures into a point system for hospital death using administrative data. Med Care. 2009;47(6):626-633. doi:10.1097/MLR.0b013e31819432e5 19433995

[16] Congress.gov. Sen. Isakson J [R G. S.2372 - 115th Congress (2017-2018): VA MISSION Act of 2018. June 6, 2018. Accessed May 20, 2025. https://www.congress.gov/bill/115th-congress/senate-bill/2372

[17] Dabbas N, Adams K, Pearson K, Royle G. Frequency of abdominal wall hernias: is classical teaching out of date? JRSM Short Rep. 2011;2(1):5. doi:10.1258/shorts.2010.010071 21286228 PMC3031184

[18] Ma Q, Jing W, Liu X, Liu J, Liu M, Chen J. The global, regional, and national burden and its trends of inguinal, femoral, and abdominal hernia from 1990 to 2019: findings from the 2019 Global Burden of Disease Study, a cross-sectional study. Int J Surg. 2023;109(3):333-342. doi:10.1097/JS9.0000000000000217 37093073 PMC10389329

[19] Neumayer LA, Gawande AA, Wang J, ; CSP #456 Investigators. Proficiency of surgeons in inguinal hernia repair: effect of experience and age. Ann Surg. 2005;242(3):344-348. doi:10.1097/01.sla.0000179644.02187.ea 16135920 PMC1357742

[20] Mills J, Liebert C, Wren SM, Pratt JSA, Earley M, Eisenberg D. Robotic general surgery trends in the Veterans Health Administration, community practice, and academic centers from 2013 to 2021. JAMA Surg. 2023;158(5):552-554. doi:10.1001/jamasurg.2022.7728 36790771 PMC9932937

[21] Ehlers AP, Rob F, Thumma J, . Comparative outcomes of groin hernia repair by sex among Medicare beneficiaries. Ann Surg. 2023;278(4):e835-e839. doi:10.1097/SLA.0000000000005794 36727846 PMC10354208

[22] US Census Bureau. American Community Survey S2101: Veteran Status. Accessed August 22, 2025. https://data.census.gov/table/ACSST1Y2021.S2101?q=S2101

[23] HerniaSurge Group. International guidelines for groin hernia management. Hernia. 2018;22(1):1-165. doi:10.1007/s10029-017-1668-x 29330835 PMC5809582

[24] Beadles CA, Meagher AD, Charles AG. Trends in emergent hernia repair in the United States. JAMA Surg. 2015;150(3):194-200. doi:10.1001/jamasurg.2014.1242 25564946

[25] Rose L, Mattingly AS, Morris AM, Trickey AW, Ding Q, Wren SM. Surgical procedures in Veterans Affairs hospitals during the COVID-19 pandemic. Ann Surg. 2021;273(4):e129-e131. doi:10.1097/SLA.0000000000004692 33351471 PMC7959859

[26] Gomez D, Nantais J, Telesnicki T, . A population-based analysis of the COVID-19 generated surgical backlog and associated emergency department presentations for inguinal hernias and gallstone disease. Ann Surg. 2022;275(5):836-841. doi:10.1097/SLA.0000000000005403 35081578 PMC9083314

[27] Miller DR, Safford MM, Pogach LM. Who has diabetes? best estimates of diabetes prevalence in the Department of Veterans Affairs based on computerized patient data. Diabetes Care. 2004;27(Suppl 2):B10-21. doi:10.2337/diacare.27.suppl_2.b10 15113777

[28] Das SR, Kinsinger LS, Yancy WS Jr, . Obesity prevalence among veterans at Veterans Affairs medical facilities. Am J Prev Med. 2005;28(3):291-294. doi:10.1016/j.amepre.2004.12.007 15766618

[29] Hoerster KD, Lehavot K, Simpson T, McFall M, Reiber G, Nelson KM. Health and health behavior differences: U.S. military, veteran, and civilian men. Am J Prev Med. 2012;43(5):483-489. doi:10.1016/j.amepre.2012.07.029 23079170

[30] Klevens RM, Giovino GA, Peddicord JP, Nelson DE, Mowery P, Grummer-Strawn L. The association between veteran status and cigarette-smoking behaviors. Am J Prev Med. 1995;11(4):245-250. doi:10.1016/S0749-3797(18)30453-7 7495601

[31] Eibner C, Krull H, Brown KM, . Current and projected characteristics and unique health care needs of the patient population served by the Department of Veterans Affairs. Rand Health Q. 2016;5(4):13. 28083423 10.7249/rhq-05-04-13 PMC5158228

[32] Odani S, Agaku IT, Graffunder CM, Tynan MA, Armour BS. Tobacco product use among military veterans - United States, 2010-2015. MMWR Morb Mortal Wkly Rep. 2018;67(1):7-12. doi:10.15585/mmwr.mm6701a2 29324732 PMC5769800

[33] Congress.gov. Rep. Rogers H [R K 5. H.R.3230 - 113th Congress (2013-2014): Veterans Access, Choice, and Accountability Act of 2014. August 7, 2014. Accessed May 20, 2025. https://www.congress.gov/bill/113th-congress/house-bill/3230

[34] Graham LA, Schoemaker L, Rose L, Morris AM, Aouad M, Wagner TH. Expansion of the Veterans Health Administration network and surgical outcomes. JAMA Surg. 2022;157(12):1115-1123. doi:10.1001/jamasurg.2022.4978 36223115 PMC9558067

[35] Apaydin EA, Paige NM, Begashaw MM, Larkin J, Miake-Lye IM, Shekelle PG. Veterans Health Administration (VA) vs. non-VA healthcare quality: a systematic review. J Gen Intern Med. 2023;38(9):2179-2188. doi:10.1007/s11606-023-08207-2 37076605 PMC10361919

[36] Blegen M, Ko J, Salzman G, . Comparing quality of surgical care between the US Department of Veterans Affairs and non-Veterans Affairs settings: a systematic review. J Am Coll Surg. 2023;237(2):352-361. doi:10.1097/XCS.0000000000000720 37154441 PMC10344435

[37] George EL, Massarweh NN, Youk A, . Comparing Veterans Affairs and private sector perioperative outcomes after noncardiac surgery. JAMA Surg. 2022;157(3):231-239. doi:10.1001/jamasurg.2021.6488 34964818 PMC8717209

[38] Harada ND, Villa VM, Andersen R. Satisfaction with VA and non-VA outpatient care among veterans. Am J Med Qual. 2002;17(4):155-164. doi:10.1177/106286060201700405 12153068

[39] Sayre GG, Neely EL, Simons CE, Sulc CA, Au DH, Michael Ho P. Accessing care through the Veterans Choice program: the veteran experience. J Gen Intern Med. 2018;33(10):1714-1720. doi:10.1007/s11606-018-4574-8 30039494 PMC6153225

[40] Tadaki C, Lomelin D, Simorov A, . Perioperative outcomes and costs of laparoscopic versus open inguinal hernia repair. Hernia. 2016;20(3):399-404. doi:10.1007/s10029-016-1465-y 26874507

[41] Jensen KK, East B, Jisova B, . The European Hernia Society Prehabilitation Project: a systematic review of patient prehabilitation prior to ventral hernia surgery. Hernia. 2022;26(3):715-726. doi:10.1007/s10029-022-02573-2 35212807

[42] Li ZW, Song M, Liu J, Jiang B, Hu W, Zheng X. Is the recurrence rate higher in obese patients undergoing inguinal hernia surgery? Hernia. 2025;29(1):107. doi:10.1007/s10029-025-03301-2 40000506

[43] Huerta S, Patel PM, Mokdad AA, Chang J. Predictors of inguinodynia, recurrence, and metachronous hernias after inguinal herniorrhaphy in veteran patients. Am J Surg. 2016;212(3):391-398. doi:10.1016/j.amjsurg.2016.01.036 27324385

[44] Marcolin P, Oliveira Trindade B, Bueno Motter S, Brandão GR, Messer N, Mazzola Poli de Figueiredo S. The impact of smoking on inguinal hernia repair outcomes: a meta-analysis of multivariable-adjusted studies. Hernia. 2024;28(4):1029-1037. doi:10.1007/s10029-024-03049-1 38717561

[45] Shahait AD, Alghanem L, Cmorej P, . Postoperative outcomes of ventral hernia repair in veterans. Surgery. 2021;169(3):603-609. doi:10.1016/j.surg.2020.09.003 33077198

[46] Howard R, Thompson M, Fan Z, Englesbe M, Dimick JB, Telem DA. Costs associated with modifiable risk factors in ventral and incisional hernia repair. JAMA Netw Open. 2019;2(11):e1916330. doi:10.1001/jamanetworkopen.2019.16330 31774525 PMC6902835

[47] Thornton MA, Marten EL, Lunardi N, . Learning how preoperative communication relates to postoperative experiences for older veterans having inguinal hernia surgery. J Am Geriatr Soc. 2026;74(1):177-185. doi:10.1111/jgs.70216 41294300 PMC13527829

[48] Jensen SAS, Lauridsen SV, Fonnes S, Rosenberg J, Tønnesen H. Effect of tailored, intensive prehabilitation for risky lifestyles before ventral hernia repair on postoperative outcomes, health, and costs: study protocol for a randomised controlled trial (STRONG-Hernia). PLoS One. 2025;20(5):e0324002. doi:10.1371/journal.pone.0324002 40435308 PMC12118980

[49] Hall DE, Arya S, Schmid KK, . Association of a frailty screening initiative with postoperative survival at 30, 180, and 365 days. JAMA Surg. 2017;152(3):233-240. doi:10.1001/jamasurg.2016.4219 27902826 PMC7180387

[50] Aydin M, Fikatas P, Denecke C, Pratschke J, Raakow J. Cost analysis of inguinal hernia repair: the influence of clinical and hernia-specific factors. Hernia. 2021;25(5):1129-1135. doi:10.1007/s10029-021-02372-1 33555463 PMC8514365

[51] Meier J, Berger M, Hogan T, . Using local rather than general anesthesia for inguinal hernia repair may significantly reduce complications for frail veterans. Am J Surg. 2021;222(3):619-624. doi:10.1016/j.amjsurg.2021.01.026 33504434 PMC8295403

[52] Fitzgibbons RJ Jr, Forse RA. Clinical practice: groin hernias in adults. N Engl J Med. 2015;372(8):756-763. doi:10.1056/NEJMcp1404068 25693015

[53] Lockhart K, Dunn D, Teo S, . Mesh versus non-mesh for inguinal and femoral hernia repair. Cochrane Database Syst Rev. 2018;9(9):CD011517. doi:10.1002/14651858.CD011517.pub2 30209805 PMC6513260

[54] Melkemichel M, Bringman SAW, Widhe BOO. Long-term comparison of recurrence rates between different lightweight and heavyweight meshes in open anterior mesh inguinal hernia repair: a nationwide population-based register study. Ann Surg. 2021;273(2):365-372. doi:10.1097/SLA.0000000000003219 30720504

[55] Bakker WJ, Aufenacker TJ, Boschman JS, Burgmans JPJ. Lightweight mesh is recommended in open inguinal (Lichtenstein) hernia repair: a systematic review and meta-analysis. Surgery. 2020;167(3):581-589. doi:10.1016/j.surg.2019.08.021 31672519

[56] Fink T, Chen Q, Chong L, Hii MW, Knowles B. Telemedicine versus face-to-face follow up in general surgery: a randomized controlled trial. ANZ J Surg. 2022;92(10):2544-2550. doi:10.1111/ans.18028 36069322 PMC9826044

[57] Rosenberg J, Christoffersen MW, Krogsgaard M, . Non-operative considerations in relation to groin and ventral hernia repair: local consensus recommendations from the Danish Hernia Database. Hernia. 2025;29(1):186. doi:10.1007/s10029-025-03377-w 40418367

[58] Bhardwaj P, Huayllani MT, Olson MA, Janis JE. Year-over-year ventral hernia recurrence rates and risk factors. JAMA Surg. 2024;159(6):651-658. doi:10.1001/jamasurg.2024.0233 38536183 PMC10974689

[59] Bay-Nielsen M, Kehlet H, Strand L, ; Danish Hernia Database Collaboration. Quality assessment of 26,304 herniorrhaphies in Denmark: a prospective nationwide study. Lancet. 2001;358(9288):1124-1128. doi:10.1016/S0140-6736(01)06251-1 11597665

[60] Köckerling F, Maneck M, Günster C, Adolf D, Hukauf M. Comparing routine administrative data with registry data for assessing quality of hospital care in patients with inguinal hernia. Hernia. 2020;24(1):143-151. doi:10.1007/s10029-019-02009-4 31342203

