# Supplementary material for: Evolving Inguinal Hernia Repair Practice at the Veterans Health Administration
Source: JAMA Surg. 2026 Mar 18;161(5):518–26. doi: 10.1001/jamasurg.2026.0307 (PMC13000745; doi:10.1001/jamasurg.2026.0307)
Supplement: Supplement 1. — eTable 1. Crosswalk of CPT and ICD codes for inguinal hernia repairs by category eTable 2. Evidence-Based Interventions to Improve Quality and Reduce Recurrence After Inguinal Hernia Repair: Potential Applications Within the Veterans Health Administration [file jamasurg-e260307-s001.pdf]

Supplemental Online Content

Bradley EM, Schlosser KA, Matheny ME, Pierce RA. Evolving inguinal hernia repair practice at the Veterans Health Administration. *JAMA Surg*. Published online March 18, 2026. doi:10.1001/jamasurg.2026.0307

eTable 1. Crosswalk of CPT and ICD codes for inguinal hernia repairs by category

eTable 2. Evidence-based interventions to improve quality and reduce recurrence after inguinal hernia repair

This supplemental material has been provided by the authors to give readers additional information about their work.

© 2026 Bradley EM et al. *JAMA Surgery*.

eTable 1. Crosswalk of CPT and ICD codes for inguinal hernia repairs by category

Hernia Type		CPT	ICD-9	ICD-10	
**Inguinal**	MIS	49650<br>49651	*Lap/open not captured<br>53.00<br>53.01<br>53.02<br>53.03<br>53.04<br>53.05<br>53.10<br>53.11<br>53.12<br>53.13<br>53.14<br>53.15<br>53.16<br>53.17<br>53.21<br>53.29<br>53.31<br>53.39	*ICD-10-PCS 0YQ54ZZ*<br>*ICD-10-PCS 0YQ64ZZ*<br>*ICD-10-PCS 0YQA4ZZ*<br>*ICD-10-PCS 0YUA47Z*<br>*ICD-10-PCS 0YUA4JZ*<br>*ICD-10-PCS 0YUA4KZ*<br>*ICD-10-PCS 0YU647Z*<br>*ICD-10-PCS 0YU64JZ*<br>*ICD-10-PCS 0YU64KZ*<br>*ICD-10-PCS 0YU747Z*<br>*ICD-10-PCS 0YU74JZ*	*ICD-10-PCS 0YU74KZ*<br>*ICD-10-PCS 0YU847Z*<br>*ICD-10-PCS 0YU84JZ*<br>*ICD-10-PCS 0YU84KZ*<br>*ICD-10-PCS 0YQ74ZZ*<br>*ICD-10-PCS 0YQ84ZZ*<br>*ICD-10-PCS 0YUE47Z*<br>*ICD-10-PCS 0YUE4JZ*<br>*ICD-10-PCS 0YUE4KZ*<br>*ICD-10-PCS 0YQE4ZZ*
	Open	49505<br>49507<br>49520<br>49521<br>49525<br>49550<br>49553<br>49555<br>49557		*ICD-10-PCS 0YQ50ZZ*<br>*ICD-10-PCS 0YQ53ZZ*<br>*ICD-10-PCS 0YQ60ZZ*<br>*ICD-10-PCS 0YQ63ZZ*<br>*ICD-10-PCS 0YU507Z*<br>*ICD-10-PCS 0YU50JZ*<br>*ICD-10-PCS 0YU50KZ*<br>*ICD-10-PCS 0YU607Z*<br>*ICD-10-PCS 0YU60JZ*<br>*ICD-10-PCS 0YU60KZ*<br>*ICD-10-PCS 0YQA0ZZ*<br>*ICD-10-PCS 0YQA3ZZ*<br>*ICD-10-PCS 0YUA07Z*<br>*ICD-10-PCS 0YUA0JZ*<br>*ICD-10-PCS 0YUA0KZ*	*ICD-10-PCS 0YU707Z*<br>*ICD-10-PCS 0YU70JZ*<br>*ICD-10-PCS 0YU70KZ*<br>*ICD-10-PCS 0YU807Z*<br>*ICD-10-PCS 0YU80JZ*<br>*ICD-10-PCS 0YU80KZ*<br>*ICD-10-PCS 0YQ70ZZ*<br>*ICD-10-PCS 0YQ73ZZ*<br>*ICD-10-PCS 0YQ80ZZ*<br>*ICD-10-PCS 0YQ83ZZ*<br>*ICD-10-PCS 0YUE07Z*<br>*ICD-10-PCS 0YUE0JZ*<br>*ICD-10-PCS 0YUE0KZ*<br>*ICD-10-PCS 0YQE0ZZ*<br>*ICD-10-PCS 0YQE3ZZ*
	HCPCS S2900 code used to identify robotic inguinal hernia repairs				

© 2026 Bradley EM et al. *JAMA Surgery*.

eTable 2. Evidence-based interventions to improve quality and reduce recurrence after inguinal hernia repair

Strategic Domain	Evidence-Based Intervention	Evidence Supporting Impact	Implementation in VA System	References
**Preoperative Optimization**	**Smoking cessation program** (minimum 4 weeks preoperatively)	Reduce postoperative complications and recurrence rates; smoking is an independent predictor of recurrence and inguinodynia	Utilize existing VA tobacco cessation resources; integrate referral into surgical preparation pathway; check cotinine level preoperatively; delay elective surgery until cessation achieved	41,43,44
	**Weight loss intervention** for obese patients (BMI >35)	BMI >35 increases risks of complications, recurrence and readmissions	Utilize VHA weight management and MOVE! programs as part of preoperative optimization for elective repairs; establish preoperative bariatric medicine consultation pathway.	41,45,46
	**Diabetes optimization** (glycemic control, insulin management)	Reduce surgical site infections and associated recurrence; in a cohort of 20,000 patients diabetes contributed to $5 million in additional spending due to readmissions & complications	Create multidisciplinary diabetes optimization protocol with primary care and endocrinology; target standardized HbA1c threshold <7% before elective surgery.	46
	**Comprehensive SNAP prehabilitation** (Smoking, Nutrition, Alcohol, Physical activity)	Address co-existing risk factors; reduce 30-day complications requiring treatment; improve long-term outcomes	Implement tailored 4-6 week prehabilitation program addressing multiple risk factors simultaneously; utilize VA's integrated care model	41,48
	**Risk stratification** and shared decision-making	Risk-adjusted counseling improves patient selection and expectation management. Frailty screening has been	Incorporate standardized risk calculators and frailty screening into VHA surgical consult workflows	49,51

© 2026 Bradley EM et al. *JAMA Surgery*.

Strategic Domain	Evidence-Based Intervention	Evidence Supporting Impact	Implementation in VA System	References
		associated with reduced surgical mortality		
	**Target high-risk patients for intensive intervention**	25% reduction in complications could save $3.6 million; 25% reduction in readmissions could save $6 million per cohort	Risk-stratify patients; allocate resources to those with multiple modifiable risk factors; measure cost savings	46
	**Enhanced preoperative counseling** focusing on realistic recovery expectations	Improve patient satisfaction; reduce anxiety about postoperative symptoms; better align expectations with reality	Train surgeons in patient-centered communication; provide standardized educational materials; address postoperative symptom expectations	47
**Surgical Technique**	**Laparoscopic/ minimally invasive approach** for appropriate candidates	Lower complication rates, decreased mortality, shorter hospital stays	Expand laparoscopic & robotic capabilities across VA facilities; provide advanced training; establish patient selection criteria	45
	**Standardized mesh placement and selection**	Optimize recurrence prevention; mesh repair is superior in terms of recurrence, some evidence supports light-weight mesh for open repairs and heavy-weight for MIS	Develop VA formulary for mesh products; create evidence-based guidelines for mesh type and location aligned with international hernia society guidelines; ensure consistent technique	52–55
**Postoperative Management**	**Surgical site infection prevention bundle**	Break the recurrence cycle—SSIs double the risk of recurrence and lead to reoperation in 30-34% of cases	Implement comprehensive SSI prevention protocol; early detection and aggressive treatment; standardized wound care	58
	**Structured patient education regarding activity modification and symptom recognition**	Reduce postoperative pain, decrease seroma formation, improve patient satisfaction and recovery	Provide individualized written instructions; recommend 2-4 week restriction on sports/heavy lifting; Leverage VHA patient education resources and digital platforms to deliver	57

© 2026 Bradley EM et al. *JAMA Surgery*.

Strategic Domain	Evidence-Based Intervention	Evidence Supporting Impact	Implementation in VA System	References
			standardized postoperative instructions	
	**Standardized postoperative follow-up** and early complication detection	Early identification of complications may reduce recurrence and need for reoperation	Utilize telehealth follow-up and secure messaging to ensure timely postoperative evaluation	56
**Emergency Prevention**	**Early elective repair strategy** to prevent emergent operations	Prevent emergency repairs with higher mortality; emergency operations are significant cost drivers	Reduce wait times for elective hernia repair; prioritize symptomatic hernias; educate patients on incarceration warning signs	50
**Quality Infrastructure**	**VA-wide hernia registry with mandatory reporting and feedback**	Enable quality improvement, outcome tracking, and research; address current 37.5% compliance issue	Establish centralized prospective database; expand structured hernia-specific data capture within the VHA CDW to support longitudinal outcomes analysis; mandate entry for all hernia repairs; provide surgeon-level feedback; conduct regular audits	59,60
**System-Level Strategy**	**Retain hernia repairs within VA system** rather than community care referrals	Maintain quality (VA surgical care equal or better than non-VA); avoid $1.38 billion cost if all repairs outsourced; reduce length of stay	Invest in VA surgical capacity and expertise; demonstrate quality equivalence or superiority to private sector	36

© 2026 Bradley EM et al. *JAMA Surgery*.
