# Supplementary material for: Evolving Inguinal Hernia Repair Practice at the Veterans Health Administration
Source: JAMA Surg. 2026 Mar 18;161(5):518–26. doi: 10.1001/jamasurg.2026.0307 (PMC13000745; doi:10.1001/jamasurg.2026.0307)
Supplement: Supplement 2. — Data sharing statement [file jamasurg-e260307-s002.pdf]

Data Sharing Statement

Bradley. Evolving Inguinal Hernia Repair Practice at the Veterans Health Administration. *JAMA Surg*. Published March 18, 2026. doi:10.1001/jamasurg.2026.0307

Data
Data available: No

Additional Information
Explanation for why data not available: Veteran data is protected under HIPAA and VHA data use policies. Access to the data is therefore restricted to authorized personnel within the VHA under approved research protocols.
